# Biopsychosocial and Environmental Correlates of Children’s Motor Competence: An Exploratory Study

**DOI:** 10.1186/s40798-024-00763-z

**Published:** 2024-08-26

**Authors:** Beatrix Algurén, Yiling Tang, Chelsea Pelletier, Patti-Jean Naylor, Guy Faulkner

**Affiliations:** 1 Department of Food and Nutrition, and Sport Science, Faculty of Education, University of Gothenburg, Gothenborg, Sweden; 2 School of Kinesiology, Faculty of Education, University of British Columbia, Vancouver, BC Canada; 3 School of Health Sciences, University of Northern British Columbia, Prince George, BC Canada; 4 School of Exercise Science, Physical and Health Education, University of Victoria, Victoria, BC Canada

**Keywords:** Motor Competence, Fundamental Movement Skills, Physical Activity, Self-perception, Biopsychosocial-ecological, School Ground Quality, Parenting, Playing Outside

## Abstract

**Background:**

Given the significance of motor competence (MC) for healthy development and as a cornerstone for lifelong physical activity (PA), it is crucial to understand the manifold factors that are associated with MC. Thus, the aim of the present study was to investigate correlates of children’s MC and their fundamental movement skills (FMS) within their daily life from a comprehensive biopsychosocial-ecological perspective.

**Methods:**

This is a cross-sectional sub-study of the ‘Physical Literacy for Communities (PL4C)’ WAVES cohort study conducted in the West Vancouver School District, Canada. Motor competence was assessed using the PLAYfun tool including overall MC score and five FMS category scores, namely, running, locomotor skills, upper and lower body control and balance skills. By means of structural equation modeling (SEM), direct associationswith MC and with the specific FMS categories addressing physical activity behavior, self-perceived physical literacy, parenting, and school ground design were investigated.

**Results:**

A total of 355 children with a mean age of 7.5 years and 111.1 min of MVPA per day participated. The group comprised 51% boys and 47% girls from 14 elementary schools. Most children were at an emerging MC-level (71%), while those at a competent MC-level exhibited significantly more daily minutes of MVPA (123 versus 109, p = 0.001). Additionally, they played outdoors more frequently and engaged in more instructor-led PA. The results revealed that logistical support from parents had not only a direct positive association with overall MC, both for girls and boys, but also with most of the FMS categories. However, the correlates of MC varied between genders and showed different patterns across the five FMS categories. While time spent in sports or coach-/instructor-led physical activities had a significant SEM generated direct effect only for boys’ MC and for locomotor, upper body object control and balance, the aesthetic design of the school grounds was only associated with girls’ MC and those same three FMS categories. Multivariate SEM could explain 26% of variance for girls’ MC and 30% for boys’.

**Conclusions:**

This exploratory baseline assessment revealed parental logistical support as an important correlate of MC, irrespective of gender. There were distinct gender patterns across biopsychosocial-ecological correlates influencing MC and FMS. Despite the heterogeneity of the results, our findings indicate a potential role of school ground design in supporting the development of children’s MC, especially for girls.

**Supplementary Information:**

The online version contains supplementary material available at 10.1186/s40798-024-00763-z.

## Key Points

• The influence of biopsychosocial-ecological correlates on motor competence (MC) varied based on gender and the specific fundamental movement skill (FMS) categories.

• While parental logistical support demonstrated a positive direct association with both genders’ MC and most of the FMS categories, coach-led physical activities appeared to enhance only boys’.

• The positive direct association of outdoor play on boys’ MC and of school ground aesthetics on girls’ MC might suggest the role of the physical environment in MC development.

**Supplementary Information:**

The online version contains supplementary material available at 10.1186/s40798-024-00763-z.

## Background

Positive effects of physical activity (PA) on health and well-being are undoubted [1–3] and there has long been recognition that motor competence (MC) is a critical element in the pathway to PA and overall health [4, 5]. Since Stodden et al. (2008) [4] provided a foundational model to explain the developmental relationships and pathways between MC, PA, self-perceived motor competence and health related fitness, research about these relationships has grown. Within pediatrics, there is a longstanding tradition of monitoring motor development as a crucial part of a child’s healthy development [6–8]. Motor competence describes goal-directed human movement and the mastery of physical skills and movement patterns for enabling participation in physical activity [9]. Motor competence is influenced and dependent on different movement skills, often described as fundamental movement skills (FMS), typically organized into three main domains; (a) locomotor, (b) object control and (c) stability) [10, 11]. Some measures of MC assess various categories of FMS within the three main domains, e.g., distinguishing locomotor into running and hopping, object control into lower- (e.g. kicking) and upper- (e.g. throwing and catching) body control, and stability including balance [7, 12–14]. During the last decade, a discourse has been growing to include further categories of FMS like cycling and swimming and to broaden the term from fundamental to foundational skills [15]. A recent systematic review revealed that not all the pathways proposed by Stodden et al.’s model have been consistently confirmed by research and identified a need for more longitudinal studies [16]. Strong evidence was found for the positive pathway from PA to MC and for MC to PA with health-related fitness as a mediator [16]. Further, there was strong positive evidence for the pathway from MC to health-related fitness but not the reverse, with locomotor skills as the specific domain most evidently associated positively with fitness and MC. The hypothesised positive relationship between MC and PA was more recently supported by a systematic review and meta-analysis of 61 studies including 22 256 adolescents (*r* = 0.20 to 0.26) [17].

Meta-analysis of movement skills intervention studies with children aged 5–11 years has also provided evidence of a positive association between MC and PA [18]. Specifically, movement skills interventions resulted in an increase in MVPA of between 13.3 and 15.7 min/day. But the results should be interpreted with caution as only five out of 19 studies included explicitly measures of FMS and 47% of studies were assessed to have a moderate to serious risk of bias [18]. In studies targeting the early years of childhood (e.g. ages 3–6 years), a significant relationship between MC and PA was likewise demonstrated [8]. While the authors found some evidence that PA was a predictor for MC, they identified the need for more longitudinal studies throughout mid to late childhood in order to gain an in-depth understanding of the reciprocal relationship and mediation effects [8].

Beside the positive impact of PA and MC on physical health, a growing body of evidence confirms its positive relationship with mental health, cognitive, and social-emotional outcomes [17, 19–21]. In a recent review, Hill et al. proposed a conceptual model outlining pathways from MC to PA and to domains of cognition and social-emotional health, reflecting a more comprehensive biopsychosocial-ecological perspective [22]. While MC may mediate participation in PA through self-perception and physical fitness, it also mediates through its impact on behavioral, neurobiological, cognitive and social-emotional health outcomes [22, 23]. These pathways are influenced by moderators, including individual characteristics such as age, sex, maturation (i.e. biological); environmental characteristics such as home, school, socio-economic status, culture (i.e. socio-ecological); and task characteristics that can be both quantitative and qualitative [22, 23]. In their analysis of 49 studies (15 observational and 34 experimental) focusing on children primarily aged 3–9 years, Hill et al. concluded that both experimental and observational studies provided some support for the effect of MC on cognitive functions [22]. However, evidence for domain-specific relationships is still not clear. Commonly across the pathway studies, authors identified the need for more robust longitudinal studies, more adequate assessment of MC and FMS, studies that account for biological maturation and more rigorous methodology that considers the complexity of the processes and the necessity to encompass moderating and mediating influences [16, 17, 22].

Considering the importance of MC for healthy development and as a foundation for lifelong PA, understanding the correlates of MC is crucial. In line with Hill et al.’s call to action [22], the aim of the present study was to increase knowledge about correlates of children’s MC from a comprehensive biopsychosocial-ecological perspective. More specifically, this study of elementary school aged children sought to: (1) identify differences between children with emerging versus competent MC-level regarding sociodemographic, movement behaviors (i.e. playing outside, training, transport mode to and from school, device-measured PA, sport participation), self-perceived physical literacy, parenting and physical environment (i.e. neighborhood and school grounds) and (2) to assess gender-specific associations and SEM generated direct effects of these correlates on overall MC, and on five specific FMS categories (i.e. running, locomotor, upper and lower body object control, balance/stability). While the international motor development research consortium highlighted the necessity for more goal-directed and effective interventions to enhance MC [24], the focus of this study is to deepen our understanding of a broader array of correlates that influence MC and FMS within a child’s daily life.

## Methods

### Study Design and Participants

This is a cross-sectional sub-study embedded within a two-year longitudinal cohort study ‘Physical Literacy for communities (PL4C)’ referred to as the ‘WAVES study’ described elsewhere [25]. The WAVES study aimed to evaluate a school district initiative to increase children’s physical literacy (PL). Physical literacy has gained increasing importance in the discourse about how to create lifelong physically active people [26–30]. Indeed, PL is a multi-dimensional concept that embraces an integrative perspective and interconnection between body and mind including affective, physical and knowledge aspects [31–34].

In the present study, children in Grade 2 or Grade 2/3 split classrooms and their parents (*n* = 473) from all 14 elementary schools in the West Vancouver school district were invited to participate. West Vancouver is a an area with a high socio-economic status (e.g., higher average household income, higher education level compared to the British Columbia province [35]). A total of 355 children (183 boys, 166 girls, 6 non-binary, response-rate 75%) with an average age of 7.5 years (*SD* = 0.5) from all schools participated. Approximately 70% of parents (*n* = 253) were aged between 30 and 44 years, most worked full-time (63%), and almost all had at least university-level education (48.5% university-, 45% graduate-level). Most parents self-identified as Asian (46%) followed by European (29%).

### Data Collection

Table 1 provides an overview of the included variables, organized according to their respective levels of a biopsychosocial-ecological framework.

**Table 1:** Overview of the included variables according to their respective perspective

Perspective	Variables	Measured
**Biological**	Age	Chronologic (parent-reported)
	Gender	Child-reported
**Psychosocial**		
Behavioral		
	*Overall Motor Competence*	*PLAYfun tool - PLAYfun score*
	*Fundamental movement skills (FMS) categories*	
	*Running (a square; there and back; run*,* jump*,* land on 2 feet)*	*PLAYfun tool- Running score*
	*Locomotor (crossovers*,* skip*,* gallop*,* hop*,* jump)*	*PLAYfun tool - Locomotor score*
	*Object control - upper body (Overhand throw*,* strike with a stick*,* one-handed catch*,* hand dribble station)*	*PLAYfun tool - Upper Body score*
	*Object control- lower body (kick ball*,* foot dribble moving forward)*	*PLAYfun tool - Lower Body score*
	*Balance (walk heel-to-toe forward*,* walk toe-to-heel backward*,* drop to the ground and get back up*,* lift and lower)*	*PLAYfun tool - Balance score*
	Travel mode to and from school	Parent-reported
	Playing outside weekday and weekend (OutdoorWD and OutdoorWE)	Parent-reported
	Participating in sports (SportTime)	Parent-reported
	Physical activity	Actigraph GT3X + BT
Self-perception		
	Overall Physical literacy (PL) score	PLAYself - PL score (self-reported)
	Confidence to be physically active in different environments (PL Environment)	PLAYself tool- Environment score (self-reported)
	Self-efficacy and how it relates to children’s participation in physical activity (PL Self Description)	PLAYself tool - PL self-description sub-score (self-reported)
	Interest in different literacies (PL Rank of Literacy)	PLAYself tool- Ranking score (self-reported)
Social support		
	Parenting (4 sub-scores: Logistics, Modeling, Community use, Restricting)	The Activity Support Scale for Multiple Groups (ACTS-MG) (parent-reported)
**Ecological**	School environment (6 sub-scores: Design, Aesthetics, Cycling, Walking, Sport Play, Other Facilities)	Sport, Physical activity and Eating behavior: Environmental Determinants in Young People (SPEEDY) school grounds audit tool
	Neighborhood	Neighbourhood Environment Walkability Scale for Youth (NEWS-Y)

Cursive written variables are the dependent variables in the structural equation modeling

### Physical-Behavioral Correlates

#### Motor Competence and Fundamental Movement Skills

Children’s motor competence was assessed using the PLAYfun tool [40]. Children were asked to perform 18 movement skill tasks across five FMS categories, i.e., (a) running (3 tasks), (b) locomotor (5 tasks), (c) object control - upper body (4 tasks), (d) object control – lower body (2 tasks), and (e) balance, stability, and body control (4 tasks) [36]. Assessment of the performance quality is done on a continuous criterion-referenced visual analogue scale (1-100) dividing motor competence level into four stages: initial (< 25), emerging (25–49), competent (50–74), and proficient (75–100). A score can be calculated for each of the five FMS categories, as well as for overall MC across all five FMS categories. In the present sample, the minimum and the maximum of the PlayFun overall score were 29.67 and 60.38 with a mean of 45.79, i.e. children were either on emerging or on competent level. Thus, for the upcoming analysis, the overall MC level of the study population was dichotomized into (1) emerging (< 50) and (2) competent ( > = 50).

The PLAYfun tool has been shown to be a valid and reliable measurement tool for measuring overall MC and specific categories of FMS within the concept of physical literacy (PL) in children and youth [37]. Factor analysis confirmed the five factor model with fit indices for RMSEA of 0.055; 90% confidence interval, 0.03–0.075; CFI, 0.95; TLI, 0.94 [13]. Convergent validity of the PLAYfun for MC and the five FMS categories was confirmed by moderate-to-large correlations (*r* = 0.40 to 0.57) with the Canadian Agility and Movement Skill Assessment (CAMSA) instrument (*r* = 0.40 to 0.57), and generally moderate correlations with the self-reported Physical Activity Questionnaire for Children (PAQ-C) (*r* = 0.30 to 0.40), with the exception of ‘balance’ which had a small correlation with both instruments (*r* = 0.24 for CAMSA and *r* = 0.18 for PAQ-C) [37]. Several studies have shown good to excellent inter-rater reliability of the PLAYfun tool, in particular for the overall MC score (ICC = 0.87–0.90) [37–39], while the internal consistency with two raters for average measures within each FMS category varied and showed moderate to good (e.g. ICC = 0.55 for balance) [37, 39]. In the present study, the inter-rater reliability was acceptable to very good for overall MC and for average measures across the five FMS categories (ICC = 0.72–0.87 between the two research assistants and ICC = 0.81–0.89 between the two PL specialists) [25]. For more information about the assessment in the present study, see Tang et al. [25].

#### Physical Activity

Travel mode to and from school was reported by parents asking about the usual transport mode (walk alone, walk with a friend/adult, cycling, school bus, public transport, and driven in vehicle). Parents also reported how many hours their child played outside on weekends and weekdays with the following response categories: ‘none at all’, ‘less than 1 hour’, ‘1 to less than 2 hours’, ‘2 to less than 3 hours’, ‘3 to less than 4 hours’, ‘4 to less than 5 hours’, ‘5 hours or more’. Additionally, parents reported the amount of time that their child engaged in sports or coach-/instructor-led physical activities during the last 7 days from ‘did not participate’, ‘less than 1 hour’, ‘1 hour to less than 3 hours’, ‘3 hours to less than 5 hours’, ‘5 hours to less than 7 hours’, ‘7 hours to less than 10 hours’, to ‘10 hours or more’. Children’s device-based physical activity was measured using Actigraph GT3X + BT (ActiGraph, Pensacola, FL) as described by Tang et al. [25]. Children were instructed to wear the triaxial accelerometer on their non-dominant wrist for seven consecutive days (except for water-based activities) with raw accelerations recorded at a 30 Hz sampling rate. To classify different PA intensities, the Chandler’s cut points [40] based on vector magnitude with an epoch lengths of 5-s were used, with the following categories: ≤305 (sedentary), 306–817 (LPA; light physical activity), 818–1968 (MPA; moderate physical activity), ≥ 1969 (VPA; vigorous physical activity).

### Psychosocial Correlates

#### Self-Perceived Physical Literacy

To reflect psychosocial factors, data collected with the PLAYself tool were used. The PLAYself tool consists of three sub-scores, (1) children’s self-confidence to do sports and activities in different physical environments (environment score), (2) statements about doing sports and activities aimed to measure child’s self-efficacy for participation in PA and referred to as PL self-description score, and (3) children’s ranking of importance of 3 literacies (i.e., a) reading and writing, b) mathematics and numbers, and c) movement, activities and sport) in school, at home with family, and with friends (ranking score). The PL self-description score was used to assess the child’s self-efficacy and its connection to their engagement in PA. Self-efficacy is the individual’s belief in their capacity to succeed in various situations [41]. The total PLAYself score is the average across the sub-scores. The higher the score (0–100), the higher the self-perceived PL. The PLAYself tool has shown excellent test-retest reliability (ICC = 0.81–0.84) and good-excellent internal consistency (PSI = 0.70–0.82) [42]. To obtain a nuanced picture of possible correlates, the three sub-scores Environment, PL Self-description and Rank of Literacy were used for analysis with structural equation modeling (SEM).

#### Parental Support

The Activity Support Scale for Multiple Groups (ACTS-MG) [43] includes four parenting factors of logistic support (i.e., enrolling child in sports, taking child to places to be physically active, watching the child playing sports or any PA), modeling (i.e., encouraging child through leading by example, regularly and enjoying exercising by themselves), use of community resources to promote physical activity (i.e. encouraging use of resources in the neighbourhood to be active, enrolling child in community based programs, finding ways for the child to be active when after school), and restriction of sedentary behaviors related to screen-time (i.e. limiting time playing video games, watching TV, using the computer for others than homework). The instrument has adequate internal reliability and good construct validity, showing a model fit for the four factors of CFI = 0.94, RMSEA (90%CI = 0.05) < 0.001–0.086 for African American and 0.033–0.097 for white American parents [44, 45]. It has been repeatedly used in Canadian samples to capture parental support for PA [46, 47].

### Ecological Correlates

#### Neighborhood

Neighborhood characteristics that may influence children’s physical activity participation [48] were assessed through two subscales of the Neighbourhood Environment Walkability Scale for Youth (NEWS-Y) [49]. Parents were asked to answer seven questions regarding pedestrian/automobile traffic safety, and six questions regarding crime safety on a 4-point Likert scale (strongly disagree to strongly agree). Prior to calculating subscale-scores (i.e. average across included questions), some questions needed reverse coding; higher scores represent a more favorable environment [50, 51]. The two subscales have demonstrated good test–retest reliability and internal consistency for parents of children (intraclass correlation coefficients (ICC), and Cronbach’s alphas of 0.87 for the crime safety subscale, and 0.74 for pedestrian/automobile traffic safety [49].

#### School Ground Assessment

The physical environment of the school was assessed with the Sport, Physical activity and Eating behavior: Environmental Determinants in Young People (SPEEDY) school grounds audit tool [52]. SPEEDY consists of 44 items, where six component scores and one overall school ground quality score can be derived; ‘cycling provision’ (e.g., cycle lanes, traffic calming, school warning signs for road users), ‘walking provision’ (e.g., marked pedestrian crossings, road safety signs), ‘sports and play facility provision’ (e.g., bright markings on play surfaces, playground equipment, courts), ‘other facility provision’ (e.g., benches, picnic tables), ‘design of the school grounds’ (e.g., suitability for informal games), ‘aesthetics’ (e.g., presence of litter, murals/outdoor art, graffiti). The total ‘school physical activity suitability score’ sums the items from ‘cycling and walking provision’, ‘sport and play facilities’ and ‘design of school grounds’ but counting for the overlapping individual items only once (n = 29) [52]. Two researchers completed the assessment independently. For elementary schools, the SPEEDY audit instrument was found to have acceptable reliability (kappa score above 0.41) and good construct validity [52].

### Statistical Analysis

To investigate differences between children with emerging and competent MC, ‘active travel’ was dichotomized into (1) ‘active travel’ (bike/walk)/public transport and (2) ‘driven in vehicle’. The variables regarding time ‘playing outside’ on weekday or weekend were trichotomized into (1) not at all, (2) 30 min to less than 2 h, and (3) 2 h or more. Variables were tested for normality with Shapiro-Wilk tests. Differences between children with emerging and competent MC levels were analysed with t-test for continuous normal-distribution, Mann-Whitney U test and Chi-square test for proportions.

**Fig. 1:**
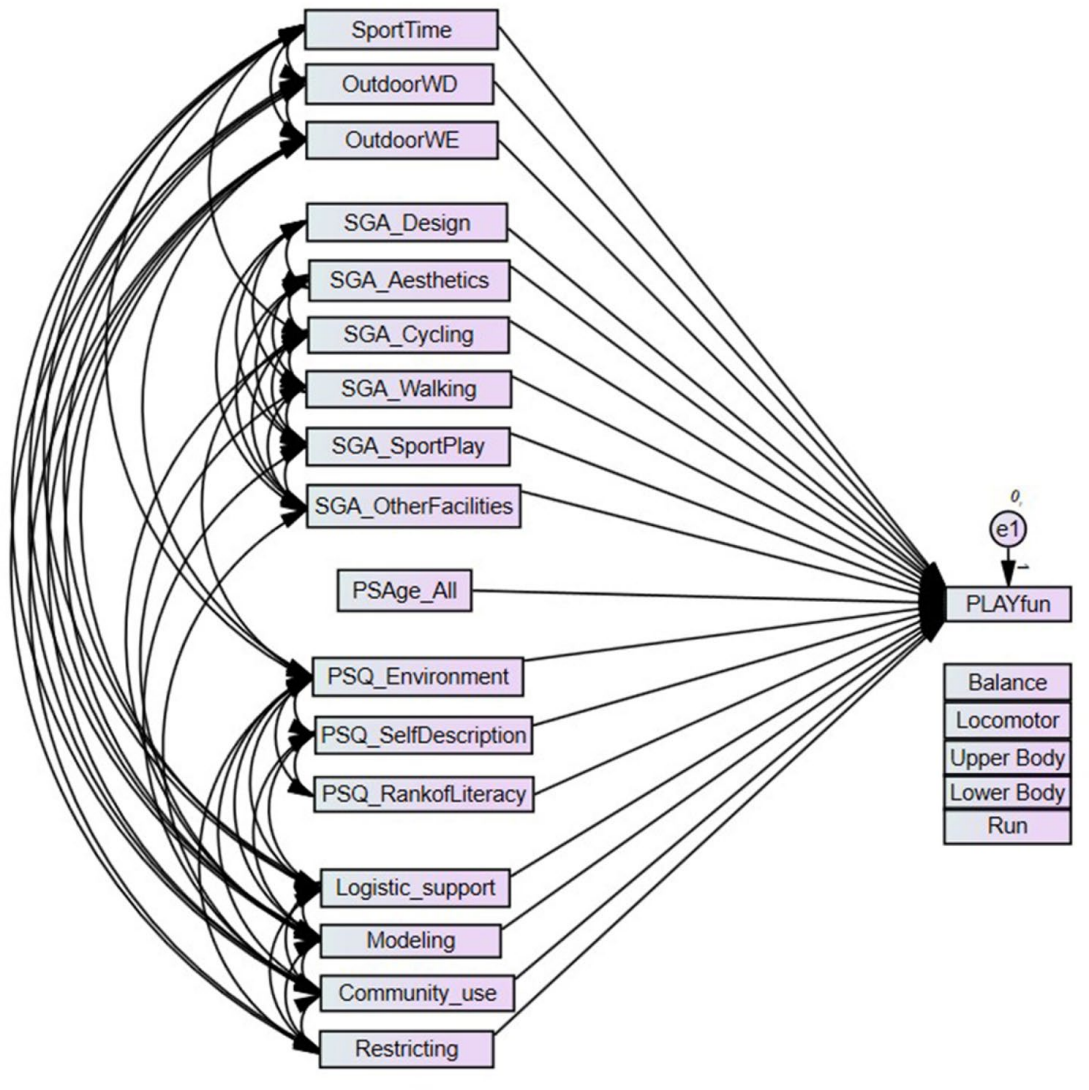
Model to explain overall motor competency, respectively the specific 5 motor competencies. Intercorrelations between the explaining variables were modelled as identified in prior univariate correlation-analysis. PSQ_Environment, PSQ_SelfDescription, PSQ_RankofLiteracy are the 3 sub-scores from the PLAYself Questionnaire. SGA_Design, SGA_Aesthetics, SGA_Cycling, SGA_Walking, SGA_SportPlay, SGA_OtherFacilities are the 6 sub-scores from the SPEEDY Questionnaire. Logistic_support, Modeling, Community_use, Restricting are the 4 sub-scores from ACTS-MG Questionnaire. SportTime = hours child participated in instructor-led activities, OutdoorWD = hours child playing outside during weekday, Outdoor WE = hours child playing outside during weekend, all three parent-reported

To assess the associations of the various biopsychosocial-ecological correlates on MC and on specific FMS categories (i.e. running, locomotor, upper and lower body object control, balance), structural equation modeling (SEM) for overall MC and each FMS category stratified by gender were processed (in AMOS ver. 29, IBM SPSS, Chicago, IL). The significant associations with overall MC and the five FMS categories identified through Pearson’s product-moment correlation for continuous normal-distributed and Spearman’s rho correlation for continuous not normal-distributed variables were the exogenous variables in the SEM. Age was included as a control variable (see Fig. 1). When describing SEM results and the calculated coefficients, the term ‘direct effect’ was used as per the SEM program.

Device-measured PA was excluded from the SEM because PA showed the highest significant correlations with MC and FMS categories as the multiple tested endogenous variables, but also with biopsychosocial ecological correlates as the exogenous variables. Together with evidence from the literature [8], we assumed a mediating effect of PA in our sample. All statistical analyses were performed in SPSS version 29 with an alpha-level of 0.05 (IBM SPSS, Chicago, IL).

Prior to SEM analysis, cases with missing data were excluded resulting in *n* = 304 complete cases. To assess model fit, Bollen-Stine bootstrapping was conducted based on 5000 bootstrapping samples [53], as univariate non-normality was observed for many included variables. A p-value larger than 0.05 was used to indicate good model fit. Additionally, several other model fit indices were consulted, including the chi-squared (χ2) test, χ2/df, comparative fit index (CFI), incremental fit index (IFI), Tucker-Lewis index (TLI), standardized root mean squared residual (SRMR), and root-mean squared error of approximation (RMSEA). The model was deemed to have excellent fit based on a non-significant χ2 value (*p* > 0.05) and a χ2/df value between 1 and 3 [54], as well as high values for CFI, IFI, and TLI (all greater than 0.95), an SRMR close to 0.08, and an RMSEA value below 0.06 with an upper 90% confidence interval (HI90) below 0.08 and a PCLOSE value above 0.5 [55]. Multigroup path analyses were conducted to assess the established model separately for boys and girls. Standardized and unstandardized coefficients were presented for all pathways, and a p-value of less than 0.05 was considered significant.

## Results

### Characteristics of Study Population

Table 1 gives an overview of study participants’ movement behaviors, social and physical environment (i.e., parenting, neighborhood and school ground quality) and PLAYself scores.

Complete assessments of MC and FMS categories (PLAYfun scores) were gathered for 319 participants (90%; 166 boys, 147 girls, 6 non-binary) with the majority being at the emerging-level of MC (79%, *n* = 238) and 22% at the competent-level (*n* = 67). There were no significant gender differences for the PLAYfun overall MC score or for the running and balance category scores. While girls scored significantly higher in the locomotor domain than boys (43 versus 41, *p* = 0.01), they scored significantly lower in the upper and lower body object control domains (43 versus 48 and 47 versus 49, *p* < 0.001). Children’s PLAYself scores were between 68 and 72 (range 0-100) on average across all three sub-scores and no gender differences were detected.

Out of the 355 children, 258 (76.8%; 128 boys, 126 girls, 4 non-binary) provided valid PA data with Actigraph GT3X + BT (i.e., met the wear time criteria). Of those, participants accumulated approximately 111 min of MVPA per day (113 min/d for boys, 109 min/d for girls, t(252) = 0.89, *p* = 0.37). Almost all participants (97.3%) met the Canadian PA guideline criteria, i.e., accumulating on average 60 min of daily MVPA. The majority of children were driven in vehicles to (72%) and from school (70%), 5% of participants took a school bus or public transport, 15% walked or biked to, and 18% from, school. There were no significant gender differences in travel mode. All children except 5 played outside each day during the week, the majority between 30 min to less than 2 h (54%), whereas during the weekend most children played outside for more than 2 h (67%). 73% of children participated in sports or coach-/instructor-led physical activities each week. Children’s social and physical environment was supportive for both boys and girls. Scores on the four subscales of the ACTS-MG ranged between 3.3 and 3.6 (range 1–4). Across the 14 schools, the overall ‘school physical activity suitability’ score ranged between 13 and 22 with a mean of 20 (±2.29). On average, all six individual school ground quality component scores reached the upper one-third of the range.

### Differences Between Participants with Emerging- and Competent Level of Motor Competency

Participants with competent-level MC were slightly but significantly older than those at emerging-level (7.6 years versus 7.4 years, *p* = 0.007). Children with competent-level MC had on average 15 min/day more MVPA compared to children with emerging-level MC (*p* = 0.001; moderate PA: t(243) = -2.724, *p* = 0.007; vigorous PA: t(243) = -4.129, *p* < 0.001). There were no significant differences in travel mode.

Children with competent-level MC spent significantly more time playing outside compared to children with emerging-level MC, both on weekdays and on the weekend (55% versus 40% weekdays, *p* = 0.036; 82% versus 65% weekends, *p* = 0.011). Children with competent-level MC were also more likely to spend at least 3 h in sports or PA with instructors than those at an emerging-level (63% versus 36%, *p* < 0.001). Regarding self-perception, there were no differences between competent- and emerging-level MC children for the Literacy ranking score, but children with competent-level MC scored significantly higher on the Environment and PL self-description score (78 versus 70, *p* < 0.001; 72 versus 66, *p* = 0.006).

There were no significant differences in the parenting of children with emerging-level MC and those at the competent-level, except for logistical support, which was slightly but significantly higher in children at competent level (3.7 versus 3.5, *p* < 0.001). There were no differences between children with competent- versus emerging-level MC in terms of neighborhood environment variables. Significant differences in school ground quality between those with competent- and emerging-level MC existed only for ‘Walking’ (4.8 versus 4.5, *p* = 0.022), ‘Other Facilities’ (4.4 versus 3.9, p = 0.014) and ‘Design’ (7.0 versus 6.8, p = 0.005)(see Table 2).

**Table 2:** Overview of study characteristics from a biopsychosocial-ecological perspective stratified by children with emerging and competent level of motor competency (measure with PLAYfun), *N* = 355

	*N*	Total, *N* = 355	Emerging MC, *n* = 252	Competent MC, *N* = 67	Statistical test
**Age (year)**, Mean (SD)	355	7.5 (0.5)	7.4 (0.52)	7.6 (0.57)	***t*(317) = -2.70**, ***p*** **= .007**
**Gender**,** n (%)**	355				Χ^2^ (1, *N* = 313) = 3.32, *p* = .071
Boys		183 (51.5)	125 (49.6)	41 (61.2)	
Girls		166 (46.8)	123 (48.8)	24 (35.8)	
Non-binary		6 (1.7)	4 (66.7)	2 (3.0)	
**Physical Activity (min/day)**,** mean (SD)**	258		191	54	
Sedentary		457.8 (57.0)	456.3 (57.2)	456.7 (54.5)	*t*(243) = − 0.055, *p* = .956
Light PA		231.2 (32.0)	230.3 (33.7)	235.8 (26.5)	*t*(243) = -1.111, *p* = .268
MVPA		111.1 (30.3)	108.3 (28.6)	122.9 (32.0)	***t*(243) = -3.216**, ***p*** **= .001**
**Playing outside - weekday**,** n (%)**	343				**Χ**^**2**^ **(1**, ***N*** = 303)* = 4.76, ***p*** **= .036**
Not at all		5 (1.4)	4 (1.7)	0 (0)	
30 min to less than 2 h		192 (54.1)	141 (58.8)	30 (44.8)	
2 h or more		146 (41.1)	95 (39.6)	37 (55.2)	
**Playing outside - weekend**,** n (%)**	343				**Χ**^**2**^ **(1**, ***N*** = 306)* = 6.91, ***p*** **= .011**
Not at all		2 (0.6)	1 (0.4)	0 (0)	
30 min to less than 2 h		104 (29.3)	83 (34.7)	12 (17.9)	
2 h or more		237 (66.8)	156 (65.3)	55 (82.1)	
**Participating in sports or PA with coach/instructor**,** n (%)**	343				**Χ**^**2**^ **(2**, ***N*** **= 308) = 17.31**, ***p*** **< .001**
Not at all		85 (23.9)	68 (28.2)	7 (10.4)	
Less than 3 h per week		122 (34.4)	87 (36.1)	18 (26.9)	
3 h or more per week		136 (38.3)	86 (35.7)	42 (62.7)	
**Travel mode to school**,** n (%)**	342				Χ^2^ (1, *N* = 306) = 0.15, *p* = .86
Active (bike/walk)/public transport		71 (20.0)	48 (20.1)	12 (17.9))	
Driven in vehicle		271 (76.3)	191 (79.9)	55 (82.1)	
**Travel mode from school**,** n (%)**	342				Χ^2^ (1, *N* = 306) = 0.001, *p* = 1.00
Active (bike/walk)/public transport		78 (22.0)	54 (22.6)	15 (22.4)	
Driven in vehicle		264 (74.4)	185 (78.1)	52 (77.6)	
					Χ^2^ (1, *N* = 283) = 0.051, *p* = 1.00
Active travel to and from school		62 (18.1)	42 (19.0)	11 (17.7)	
Driven to and from school		255 (74.6)	179 (81.0)	51 (82.3)	
**Parental activity support**,** mean (SD)**	342		240	67	
Logistic		3.5 (0.54)	3.5 (0.53)	3.7 (0.52)	***U*** **= 10170.50**, ***p*** **< .001**
Modeling		3.3 (0.6)	3.3 (0.57)	3.3 (0.72)	*U* = 8997.50, *p* = .125
Community use		3.4 (0.55)	3.4 (0.52)	3.4 (0.59)	*U* = 8948.50, *p* = .148
Restricting		3.6 (0.56)	3.5 (0.53)	3.6 (0.58)	*U* = 8605.50, *p* = .343
**Neighborhood**,** mean (SD)**	343				
Crime safety		3.0 (0.77)	3.0 (0.78)	3.1 (0.74)	*U* = 8550.00, *p* = .424
Neighborhood safety (pedestrian and traffic)		2.6 (0.50)	2.6 (0.48)	2.6 (0.52)	*U* = 8398.50, *p* = .575
**School Ground**,** mean (SD)** **(min – max)**	355		252	67	
Cycling (4–8)		6.7 (1.08)	6.6 (1.13)	6.9 (0.93)	*U* = 9107.00, *p* = .29
Walking (2–5)		4.6 (0.89)	4.5 (0.97)	4.8 (0.58)	***U*** **= 9583.00**, ***p*** **= .022**
Sports and play facility provision (6–11)		9.0 (1.52)	8.9 (1.6)	8.9 (1.44)	*U* = 8322.50, *p* = .85
Other Facilities (2–6)		4.1 (1.30)	3.9 (1.27)	4.4 (1.40)	***U*** **= 10002.00**, ***p*** **= .014**
Aesthetics (20–26)		23.8 (1.67)	23.8 (1.65)	24.0 (1.5)	*U* = 9354.50, *p* = .164
Design (4–8)		6.9 (0.83)	6.8 (0.81)	7.0 (0.98)	***U*** **= 10072.00**, ***p*** **= .005**
School PA suitability (13–22)		19.9 (2.29)	19.7 (2.3)	20.2 (2.38)	*U* = 9723.50, *p* = .051
**PLAYself**,** mean (SD)**	334		251	67	
PL Environment		71.5 (15.27)	70.1 (15.45)	77.7 (12.78)	***U*** **= 10824.00**, ***p*** **< .001**
PL Self-Description		67.6 (15.63)	66.4 (15.95)	72.4 (13.44)	***U*** **= 10250.00**, ***p*** **= .006**
PL Rank of Literacy		68.7 (16.45)	68. 7 (16.33)	69.5 (17.03)	*U* = 8838.50, *p* = .52

* Group with less than *n* = 5 in cells were excluded from Chi-square test, bold text in the final column signifies *p* < .05 or more

### Correlates and SEM Generated Direct Effects of Movement Behaviors, Parenting, School Grounds Design and Perceived Physical Literacy on Overall Motor Competence

Univariate analysis revealed significant correlations of MC with parenting (ACTS-MG scores), quality of school grounds (SPEEDY scores), self-perception (PLAYself sub-scores) and movement behaviors except for travel mode (see Tables 1, 2, 3 and 4 in supplemental file). Correlations differed between girls and boys, e.g., ‘playing outdoors during weekdays’ was significantly correlated with PLAYfun MC only for boys (*r* = 0.21, *p* < 0.001), while ‘playing outdoors during weekend’ was significantly correlated only for girls (*r* = 0.23, *p* < 0.001). The correlation between MVPA and MC was stronger for boys compared to girls (*r* = 0.35 versus 0.25, *p* < 0.01). Tables 3 and 4 provides an overview of the direct effects (unstandardized and standardized) as shown by the SEM for overall MC, differentiated for boys and girls. Significant positive direct effects on boys’ MC were shown for ‘Sport/instructor-led PA’ (γ = 0.182), ‘Playing outdoors on weekdays’ (γ = 0.187), ‘Walking’ (γ = 0.239) as well as ‘Logistics’ (γ = 0.269) but a negative direct effect by ‘Cycling provision’ (γ=-0.223), together explaining 30% of variance in MC. For girls’ MC, positive significant direct effects were identified for ‘Aesthetics’ (γ = 0.210), age (γ = 0.168) and ‘Logistics’ (γ = 0.243) and these factors explained 26% of the variance in MC.

**Table 3:** Direct effects of movement behavior, social and physical environment on motor competence, *N* = 304

			Boys				Girls			
Parameter		Correlates	unstandardized estimate	standardized estimate	*p*	R^2^	unstandardized estimate	standardized estimate	*p*	R^2^
PLAYfun total score	<---	Sport time	**0.631**	**0.182**	**0.02**	**0.30**	0.096	0.031	0.716	**0.26**
PLAYfun total score	<---	Playing outdoor WD	**0.75**	**0.187**	**0.02**		0.075	0.019	0.813	
PLAYfun total score	<---	Playing outdoor WE	0.018	0.004	0.96		0.426	0.095	0.242	
PLAYfun total score	<---	Aesthetics	0.459	0.136	0.069		**0.704**	**0.21**	**0.018**	
PLAYfun total score	<---	Cycling	**-1.14**	**-0.223**	**0.041**		1.24	0.254	0.054	
PLAYfun total score	<---	Walking	**1.498**	**0.239**	**0.033**		0.179	0.032	0.813	
PLAYfun total score	<---	Sport Play	-0.319	-0.091	0.325		-0.319	-0.088	0.364	
PLAYfun total score	<---	Other Facilities	0.706	0.164	0.078		0.469	0.114	0.267	
PLAYfun total score	<---	Design	0.164	0.025	0.755		0.361	0.055	0.553	
PLAYfun total score	<---	Age	0.731	0.073	0.279		**1.677**	**0.168**	**0.02**	
PLAYfun total score	<---	PL Environment	0.059	0.162	0.09		0.063	0.173	0.068	
PLAYfun total score	<---	PL Self Description	0.041	0.125	0.207		-0.014	-0.041	0.652	
PLAYfun total score	<---	PL Rank of Literacy	-0.043	-0.143	0.054		-0.045	-0.124	0.088	
PLAYfun total score	<---	Logistics	**2.665**	**0.269**	**0.004**		**2.576**	**0.243**	**0.012**	
PLAYfun total score	<---	Modeling	-1.239	-0.156	0.071		-0.323	-0.03	0.73	
PLAYfun total score	<---	Community use	-0.557	-0.055	0.541		0.306	0.029	0.774	
PLAYfun total score	<---	Restricting	-0.358	-0.035	0.655		-0.706	-0.073	0.369	

WD = weekday, WE = weekend, PL = Physical literacy, bold text marks significant effects with *p* < .05 or more

**Table 4:** Overview of the significant direct effects from school yards, parenting, physical literacy, age on motor competencies, left girls, right boys

	Girls										Boys							
	PLAYfun	Locomotor	Upper Body	Lower Body	Balance	Running	positive	negative			PLAYfun	Locomotor	Upper Body	Lower Body	Balance	Running	positive	negative
Sport time			↑				1			Sport time	↑	↑	↑		↑		4	
Playing outdoor WD										Playing outdoor WD	↑			↑*		↑	3	
Playing outdoor WE										Playing outdoor WE								
Aesthetics	↑	↑	↑		↑		4			Aesthetics					↑*		1	
Cycling		↑*				↑	2			Cycling	↓		↓		↓			3
Walking			↑				1			Walking	↑				↑*		2	
Sport Play					↓			1		Sport Play								
Other Facilities					↑		1			Other Facilities					↑*		1	
Design										Design								
Age	↑	↑*		↓	↑		3	1		Age								
PL Environment			↑*				1			PL Environment						↑	1	
PL Self Description										PL Self Description								
PL Rank of Literacy			↓*	↓		↓		3		PL Rank of Literacy			↓	↓	↓			3
Logistics	↑*		↑*			↑	3			Logistics	↑		↑*		↑	↑	4	
Modeling										Modeling		↓				↓		2
Community use										Community use								
Restricting										Restricting								
R^2^	0.26	0.31	0.22	0.22	0.20	0.14				R^2^	0.30	0.20	0.30	0.20	0.30	0.20		

Arrows indicate significant effects, upwards = positive, downwards = negative; ↑: *p* < .05, ↑*: *p* < .001; for the size of the effect, please see Tables 3, and 5–9 in supplemental file. WD = weekday, WE = weekend; PLAYfun is the overall score for motor competence, PL = Physical literacy

### Correlates and SEM Generated Direct Effects of Movement Behaviors, Parenting, School Grounds Design and Perceived Physical Literacy on Fundamental Movement Skill Categories

All five FMS categories correlated significantly with MVPA, however, the strength of correlation differed between boys and girls, with stronger correlations for boys ranging from *r* = 0.21 to 0.40 for boys (no significance for *Balance*) versus to *r* = 0.14 to 0.25 for girls (no significance for *Locomotor* and *Balance*)(see Table 1 supplemental file).

Tables 5, 6, 7, 8 and 9 in the supplemental files provides an overview of the direct effects (unstandardized and standardized) as shown by the SEM for each FMS category, differentiated for boys and girls. While the greatest amount of variance in FMS categories could be explained for girls’ *Locomotor* (R^2^ = 0.31), and boys’ *Upper Body Object Control* and *Balance* (R^2^ = 0.30), the least could be explained by girls’ *Running* (R^2^ = 0.14). The number of significant correlates varied between the five FMS categories and across gender. For example, seven out of 15 correlates in the model turned significant for explaining boys’ *Balance*, whereas only four correlates for girls’ with ‘Other Facilities’ as the strongest direct effect for both genders (γ = 0.347, *p* < 0.001 respectively (γ = 0.320, *p* = 0.003). On the other hand, six significant correlates could explain 22% variance of girls’ *Upper Body Object Control*, though only four for boys’ explaining 30% variance with ‘Logistics’ as the strongest direct effect for both gender (γ = 0.255, p < 0.001 respective γ = 0.305, p < 0.001).

Table 4 provides an overview of the direct effects (positive or negative) of the significant correlates on overall MC and the five FMS categories, distinguished for boys and girls. ‘Sport/instructor-led PA’ and ‘Logistics’ had the most common positive effect on MC and across the five FMS categories for boys (*n* = 4), as did ‘Aesthetics’ for girls (n = 4).

## Discussion

The present study contributes to the scarce body of literature that has investigated correlates of MC, employing a SEM derived direct effect analysis to examine the biopsychosocial-ecological factors within a child’s daily life that collectively influence children’s overall MC and specific FMS categories. The present study sample, which includes physically active Canadian children, demonstrated a level of MC comparable to that of same age children in previous studies [25, 56–58]. Like recent studies [36], children who exhibited competent levels of MC were typically older, engaged in higher levels of MVPA, and were predominantly male. Our findings align with those presented in Barnett et al.’s review and meta-analysis on correlates of MC [59]. In their study, a positive association between age and MC was observed, although variations were noted when examining specific FMS categories. In the present study, children at a competent level of MC were significantly older, with no direct association with age among boys when controlling for other correlates. For girls, age was significantly associated with overall MC, locomotor and balance skills while controlling for those correlates. Given that the older children have more opportunities to engage in activities that foster skill development and that natural maturation occurs, it could be expected that MC and FMS would be greater in older children [59, 60]. Further, the amount of time playing outside, both during weekdays and at weekend, was also associated with a higher MC level. However, a direct association of playing outside weekdays was evident only for boys in relation to overall MC, lower body object control and running when investigated by SEM. While outdoor play has been consistently associated with higher physical activity levels and thereby connected to health-benefits [61–63], Lynch (2017) emphasized that outdoor play offers substantial and meaningful opportunities for motor development as well [64], which appeared to be the case for boys in our study.

In addition to playing outside, time participating in instructor-led PA/sports also had a positive impact on MC (as well on PA-level), and our results are consistent with previous research findings [59]. Our results revealed that two-thirds of children with competent MC levels spent three or more hours per week with coaches, contrasting with one third of those at the emerging MC level. The amount of time spent with coaches was associated with MC level for both genders, but no direct association persisted for girls when controlling for other correlates. The direct association remained for boys’ overall MC, and for locomotor, upper body object control and balance skills. Barnett et al.’s previous meta-analysis confirmed gender differences in the enhancement of FMS through sports activities, suggesting both gender preferences and gender normative parental support as contributing factors [59].

Our results revealed that children at a competent level of MC had higher self-confidence to be physically active in different environments and higher self-efficacy for participation in PA. However, evidence for the role of self-perception in the pathways from MC to PA and the reverse is still inconclusive [16]. In our analysis, we were able to identify a more nuanced result, indicating that feeling confident in participating in sports and activities across various environments (such as water, gym, playground, ice, snow, etc.) had a direct positive association on running for boys and on object control skills for girls. A relationship between perceived and actual MC, specifically regarding object control, was also supported by findings from a longitudinal study conducted by Strotmeyer et al. [65].

In terms of the social environment, parents often serve as the primary source of social support in early childhood. Interestingly, screen-time restrictions, and encouragement of community use for PA did not have a significant direct association on MC and the FMS categories in our study; however, the provision of logistical support was found to be significantly associated with higher levels of MC, regardless of gender or specific FMS categories, and remained when accounting for various correlates. Previous research on the impact of parental support has primarily focused on PA among children and less on children’s MCs [66–69]. In the latest meta-analysis conducted by Yao and Rhodes, encompassing 112 studies, a medium effect size was found for the relationship between overall parental support and child’s PA [66]. The analysis revealed significant heterogeneity in this relationship. Notably, parental encouragement emerged as the only social behavior with a moderate effect size. A recent study from He et al. confirmed both parental encouragement and engagement as positively associated with locomotor, ball and overall motor skills [70]. Our results align with this trend, as questions about encouragement were included in the scores for both, logistical support and community use. The impact of modeling remains uncertain; our findings showed a positive significant correlation with overall MC and locomotor exclusively in girls. However, modeling did not have a significant direct positive association when controlling for all factors, instead revealing a negative direct association with boys’ locomotor and running. Yao and Rhodes highlighted that the influence of parental support behavior on a child’s PA may vary depending on the gender of the parents [66]. In a more recent meta-analysis conducted by Su et al., which examined the effects of both positive and negative parental support (e.g., restrictions and punishment) on a child’s PA, a medium effect size for the relationship of positive parental support was also confirmed [67].

Looking closer into the associations between MC and the physical environment, specifically the design of school grounds, the results unveiled that the presence of amenities like benches, wildlife gardens, and available equipment (i.e. ‘other facilities’) had a significant direct positive association on balance for both – girls and boys. Further, school ground aesthetics (e.g. planted beds, trees) directly influenced positive girls’ overall MC and three specific FMS categories, namely locomotor, upper body control and balance; the latter as well among boys. It is worth mentioning that sport play facilities did not appear to have a direct positive association on either children’s overall MC or specific FMS categories in the present study. Possible explanations for these findings might be that all 14 schools scored consistently highly on sports play facility with a low variability between schools, and the children in the study were very physically active, with 90% meeting the Canadian guidelines for PA and around three-quarters participating in organized sports. While Jones et al. found a significant difference of nearly two minutes in boys’ MVPA-level between schools with lowest and highest scores regarding the provision of sports play facilities, they only identified a significant difference of about one minute in girls’ MVPA-level between schools with lowest and highest scores on school ground design [52]. The positive influence of school grounds and the physical environment on PA-levels, thereby potentially enhancing overall MC and specific FMS, has been confirmed by previous studies [71–73]. Our findings that characteristics of school ground design may have differential effects for movement behaviors and thereby motor development for boys and girls warrant closer attention in future research.

Overall, the results of our present study unveiled diverse patterns, contingent on gender, overall MC and specific FMS categories. Consistent with prior research, these findings support the assumption that the development of MC and FMS is related to multifaceted factors and their interplay. By collectively considering the biopsychosocial-ecological correlates in a child’s daily life, we were able to explain between 26 and 30% variance in overall MC and between 14 and 31% in FMS categories.

### Limitations

When interpreting the findings of the present study, it is important to consider some limitations (see also Tang et al. [25]). Firstly, the study population consisted of individuals from a high socioeconomic status, attending schools with favorable environments. Additionally, most of the children met the PA-guidelines. As a result, the sample may not be generalizable to other Canadian and International samples. Secondly, the design was cross-sectional which only allows identification of associations between various correlates and MC and FMS respectively with direct effects determined theoretically through SEM. To draw more robust conclusions, a longitudinal analysis of the cohort study will be conducted in the future. Thirdly, to measure overall MC and specific FMS categories we used the PLAYfun tool. The tool was originally developed for practitioners and more focused on feasibility in practice than in research. The reliability of the raters using PLAYfun in the present study was acceptable to very good. A small number of validation studies show mixed but mainly satisfactory results for its validity and reliability, especially for overall MC [37]. Further studies might be necessary to confirm its psychometric properties and our findings should be interpreted in light of this.

## Conclusions

This exploratory baseline assessment embedded within a longitudinal PL4C study provides insight into a broader range of correlates of MC and FMS, revealing distinct gender patterns across biopsychosocial-ecological factors. Parental logistical support consistently proved to be an important correlate of MC in children, regardless of gender. Furthermore, while the aesthetics of the school grounds and age were related to girls’ MC and several FMS categories, spending time engaged in instructor-led PA and engaging in free-play during weekdays were two correlates common positively associated with boy’s MC and some FMS categories. Our findings highlight the potential role of school ground design in supporting the development of children’s MC, in particular for girls. However, the study is an explorative investigation. The longitudinal nature of the WAVES study will allow for ongoing assessment of the inter-relationships between these correlates over time and will contribute to a more robust understanding of factors influencing MC and FMS development.

### Electronic Supplementary Material

Below is the link to the electronic supplementary material.

Supplementary Material 1

## Data Availability

The datasets used and/or analyzed during the current study are available from author GF on reasonable request.

## Abbreviations

ACTS-MG: Activity Support Scale for Multiple Groups
CFI: Comparative fit index
FMS: Fundamental movement skills
IFI: Incremental fit index
MVPA: Moderate to vigorous physical activity
MC: Motor competence
NEWS-Y: Neighbourhood Environment Walkability Scale for Youth
PA: Physical activity
PL: Physical Literacy
PL4C: Physical Literacy for communities
RMSEA: Root-mean squared error of approximation
SPEEDY: Sport, Physical activity and Eating behavior: Environmental Determinants in young people school grounds audit tool
SRMR: Standardized root mean squared residual
SEM: Structural equation modeling
TLI: Tucker-Lewis index
