# Supplementary material for: Biopsychosocial and Environmental Correlates of Children’s Motor Competence: An Exploratory Study
Source: Sports Med Open. 2024 Aug 26;10:90. doi: 10.1186/s40798-024-00763-z (PMC11345348; doi:10.1186/s40798-024-00763-z)
Supplement: Supplementary file 1 — Supplementary Material 1 [file 40798_2024_763_MOESM1_ESM.docx]

Supplemental file

Title:

Biopsychosocial and environmental correlates of children's motor competence – an exploratory study

Corresponding author:

Beatrix Algurén, [beatrix.alguren@gu.se](mailto:beatrix.alguren@gu.se)

Department of Food and Nutrition, and Sport Science, Faculty of Education, University of Gothenburg, Gothenborg, Sweden

Content:

Table 1. Pearson product-moment correlation between PLAYfun scores and movement behavior.

Table 2. Pearson product-moment correlation of the PLAYfun and PLAYself scores and domains.

Table 3. Spearman’s rank-order correlation between quality attributes of school grounds’ and PLAYfun scores.

Table 4. Spearman’s rank-order correlation of attributes of neighborhood, parenting and PLAYfun.

Table 5. Direct effects of movement behavior, social and physical environment on locomotor skills, N=304.

Table 6. Direct effects of movement behavior, social and physical environment on Upper Body Control, N=304.

Table 7. Direct effects of movement behavior, social and physical environment on Lower Body Control, N=304.

Table 8. Direct effects of movement behavior, social and physical environment on Balance, N=304.

Table 9. Direct effects of movement behavior, social and physical environment on Running, N=304.

Table 1. Pearson product-moment correlation between PLAYfun scores and movement behavior.

| **Pearson** |  | **PLAYfun** | **Locomotor** | **Upper Body** | **Lower Body** | **Balance** | **Running** |
| --- | --- | --- | --- | --- | --- | --- | --- |
| **Time playing outdoors - weekday (a)** | | | |  |  |  |  |
|  | Total | .165^**^ | .121^*^ | .136^*^ | .122^*^ | .130^*^ | .097 |
|  | Boys | .213^**^ | .160^*^ | .168^*^ | .170^*^ | .181^*^ | .150 |
|  | Girls | .152 | .153 | .146 | .111 | .112 | .066 |
| **Time playing outdoors – weekend (a)** | | |  |  |  |  |  |
|  | Total | .164^**^ | .114^*^ | .129^*^ | .075 | .147^**^ | .129^*^ |
|  | Boys | .133 | .105 | .126 | .008 | .172^*^ | .119 |
|  | Girls | .225^**^ | .151 | .151 | .175^*^ | .129 | .157 |
| **Time participating in sport/instructor-led PA (last 7 days)** | | | | | | |  |
|  | Total | .173^**^ | .292^**^ | .263^**^ | .221^**^ | .231^**^ | .172^**^ |
|  | Boys | .278^**^ | .238^**^ | .268^**^ | .148 | .262^**^ | .141 |
|  | Girls | .291^**^ | .367^**^ | .187^*^ | .216^**^ | .154 | .182^*^ |
| **Sedentary (min/d)** | |  |  |  |  |  |  |
|  | Total | -.008 | -.068 | .036 | .021 | -.019 | .009 |
|  | Boys | .086 | .004 | .092 | .062 | .078 | .120 |
|  | Girls | -.117 | -.117 | -.067 | -.041 | -.120 | -.087 |
| **Light PA (min/d)** | |  |  |  |  |  |  |
|  | Total | .109 | .056 | .124 | .066 | .083 | .090 |
|  | Boys | .123 | .122 | .136 | .070 | .077 | .070 |
|  | Girls | .122 | .003 | .168 | .094 | .112 | .112 |
| **MVPA** | |  |  |  |  |  |  |
|  | Total | .300^**^ | .233^**^ | .320^**^ | .263^**^ | .127^*^ | .200^**^ |
|  | Boys | .353^**^ | .335^**^ | .399^**^ | .313^**^ | .128 | .210^*^ |
|  | Girls | .245^**^ | .145 | .246^**^ | .218^*^ | .135 | .192^*^ |
| **Moderate PA** | |  |  |  |  |  |  |
|  | Total | .281^**^ | .231^**^ | .287^**^ | .232^**^ | .133^*^ | .185^**^ |
|  | Boys | .346^**^ | .332^**^ | .391^**^ | .297^**^ | .141 | .194^*^ |
|  | Girls | .229^*^ | .127 | .231^*^ | .206^*^ | .132 | .181^*^ |
| **Vigorous PA (a)** | |  |  |  |  |  |  |
|  | Total | .248^**^ | .147^*^ | .330^**^ | .258^**^ | .026 | .165^**^ |
|  | Boys | .228^*^ | .237^**^ | .310^**^ | .242^**^ | -.012 | .175 |
|  | Girls | .185^*^ | .167 | .162 | .140 | .070 | .174 |

a) Spearman rho correlation (not-normal distributed), * Correlation is significant at the 0.05 level (2-tailed), ** Correlation is significant at the 0.01 level (2-tailed)

Table 2. Pearson product-moment correlation of the PLAYfun and PLAYself scores and domains.

| **Pearson** |  |  | **PLAYfun** | **Locomotor** | **Upper Body** | **Lower Body** | **Balance** | **Running** |
| --- | --- | --- | --- | --- | --- | --- | --- | --- |
| **Environment** | | |  |  |  |  |  |  |
|  | Total |  | .310^**^ | .252^**^ | .204^**^ | .171^**^ | .103 | .146^**^ |
|  | Boys |  | .241^**^ | .252^**^ | .236^**^ | .125 | .107 | .203^**^ |
|  | Girls |  | .239^**^ | .261^**^ | .237^**^ | .267^**^ | .094 | .085 |
| **PL Self Description** | | |  |  |  |  |  |  |
|  | Total |  | .169^**^ | .154^**^ | .144^*^ | .156^**^ | .105 | .089 |
|  | Boys |  | .204^**^ | .222^**^ | .222^**^ | .137 | .086 | .122 |
|  | Girls |  | .139 | .080 | .085 | .202^*^ | .133 | .058 |
| **Rank of Literacy (a)** | | | |  |  |  |  |  |
|  | Total |  | .168^**^ | .090 | -.096 | -.062 | -.035 | -.024 |
|  | Boys |  | -.053 | .110 | -.121 | -.134 | -.089 | -.011 |
|  | Girls |  | -.006 | .053 | -.098 | -.013 | -.005 | -.077 |
| **PLAYself Score** | | |  |  |  |  |  |  |
|  | Total |  | .145^**^ | .198^**^ | .093 | .097 | .081 | .075 |
|  | Boys |  | .140 | .231^**^ | .121 | .036 | .043 | .109 |
|  | Girls |  | .148 | .152 | .081 | .197^*^ | .118 | .022 |

a) Spearman’s rank order correlation (not-normal distributed variable), * Correlation is significant at the 0.05 level (2-tailed), ** Correlation is significant at the 0.01 level (2-tailed)

Table 3. Spearman’s rank-order correlation between quality attributes of school grounds’ and PLAYfun scores.

| **Spearman** |  | **PLAYfun** | **Locomotor** | **Upper Body** | **Lower Body** | **Balance** | **Running** |
| --- | --- | --- | --- | --- | --- | --- | --- |
| **Cycling provision** | | |  |  |  |  |  |
|  | Total | .055 | .195^**^ | -.049 | .023 | -.016 | .063 |
|  | Boys | .009 | .194^*^ | -.110 | -.014 | -.106 | .067 |
|  | Girls | .124 | .209^*^ | .014 | .073 | .108 | .089 |
| **Walking provision** | |  |  |  |  |  |  |
|  | Total | .174^**^ | .134^*^ | .068 | .175^**^ | .194^**^ | .110^*^ |
|  | Boys | .217^**^ | .219^**^ | .054 | .157^*^ | .168^*^ | .189^*^ |
|  | Girls | .102 | .021 | .061 | .174^*^ | .214^**^ | .020 |
| **Sports and play facility provision** | | |  |  |  |  |  |
|  | Total | .020 | .113^*^ | -.062 | -.047 | .029 | .028 |
|  | Boys | .041 | .127 | -.116 | -.119 | .103 | .079 |
|  | Girls | .010 | .087 | .048 | .078 | -.080 | -.038 |
| **Other Facilities provision** | | |  |  |  |  |  |
|  | Total | .131^*^ | .156^**^ | .002 | .001 | .225^**^ | .097 |
|  | Boys | .164^*^ | .193^*^ | -.014 | -.025 | .286^**^ | .100 |
|  | Girls | .115 | .136 | .045 | .048 | .169^*^ | .103 |
| **Aesthetics** | | |  |  |  |  |  |
|  | Total | .072 | .028 | .109 | .062 | .058 | .014 |
|  | Boys | .027 | -.060 | .082 | .064 | .084 | -.027 |
|  | Girls | .103 | .144 | .131 | .035 | .018 | .051 |
| **Design of the school grounds** | | |  |  |  |  |  |
|  | Total | .176^**^ | .085 | .100 | .161^**^ | .181^**^ | .162^**^ |
|  | Boys | .198^*^ | .111 | .053 | .119 | .265^**^ | .186^*^ |
|  | Girls | .176^*^ | .067 | .214^**^ | .234^**^ | .103 | .150 |
| **Overall quality of school grounds** | | |  |  |  |  |  |
|  | Total | .167^**^ | .186^**^ | .042 | .109 | .155^**^ | .135^*^ |
|  | Boys | .219^**^ | .233^**^ | .001 | .062 | .240^**^ | .223^**^ |
|  | Girls | .138 | .145 | .141 | .207^*^ | .068 | .060 |

* Correlation is significant at the 0.05 level (2-tailed), ** Correlation is significant at the 0.01 level (2-tailed)

Table 4. Spearman’s rank-order correlation of attributes of neighborhood, parenting and PLAYfun.

| **Spearman** |  | **PLAYfun** | **Locomotor** | **Upper Body** | **Lower Body** | **Balance** | **Running** |
| --- | --- | --- | --- | --- | --- | --- | --- |
| **Crime Safety** | | |  |  |  |  |  |
|  | Total | .054 | .003 | .077 | -.016 | .019 | .086 |
|  | Boys | .064 | .003 | .124 | -.032 | .096 | .032 |
|  | Girls | .059 | .015 | .070 | .027 | -.037 | .147 |
| **Neighborhood Safety** | | |  |  |  |  |  |
|  | Total | .038 | -.004 | .045 | .058 | .047 | .063 |
|  | Boys | .055 | .034 | .038 | .054 | .100 | .063 |
|  | Girls | .022 | -.036 | .048 | .038 | -.029 | .052 |
| **Parenting** | |  |  |  |  |  |  |
| **Logistic Support** | | |  |  |  |  |  |
|  | Total | .304^**^ | .230^**^ | .283^**^ | .248^**^ | .201^**^ | .244^**^ |
|  | Boys | .266^**^ | .195^*^ | .298^**^ | .209^**^ | .177^*^ | .206^**^ |
|  | Girls | .347^**^ | .306^**^ | .253^**^ | .304^**^ | .230^**^ | .297^**^ |
| **Modeling** | | |  |  |  |  |  |
|  | Total | .073 | .041 | .136^*^ | .038 | .046 | .027 |
|  | Boys | -.029 | -.097 | .065 | -.001 | -.008 | -.063 |
|  | Girls | .165^*^ | .191^*^ | .157 | .088 | .081 | .116 |
| **Enhancing Community use** | | |  |  |  |  |  |
|  | Total | .158^**^ | .170^**^ | .138^*^ | .105 | .084 | .132^*^ |
|  | Boys | .038 | .065 | .049 | .031 | .003 | .091 |
|  | Girls | .278^**^ | .269^**^ | .256^**^ | .225^**^ | .146 | .175^*^ |
| **Restricting** | | |  |  |  |  |  |
|  | Total | .005 | .102 | -.011 | -.019 | -.052 | -.013 |
|  | Boys | -.048 | .057 | -.037 | -.111 | -.058 | -.061 |
|  | Girls | .082 | .153 | .041 | .123 | -.048 | .041 |

* Correlation is significant at the 0.05 level (2-tailed), ** Correlation is significant at the 0.01 level (2-tailed)

Table 5. Direct effects of movement behavior, social and physical environment on locomotor skills, N=304.

|  |  |  | Boys |  |  |  | Girls |  |  |  |
| --- | --- | --- | --- | --- | --- | --- | --- | --- | --- | --- |
| Parameter |  |  | non-stand estimate | standardized estimate | *p* | R^2^ | non-stand estimate | standardized estimate | *p* | R^2^ |
| Locomotor | <--- | Sport | **0.798** | **0.166** | **0.045** | **0.2** | 0.412 | 0.084 | 0.307 | 0.31 |
| Locomotor | <--- | Playing outdoor WD | 0.648 | 0.117 | 0.172 |  | 0.032 | 0.005 | 0.948 |  |
| Locomotor | <--- | Playing outdoor WE | 0.062 | 0.011 | 0.906 |  | 0.48 | 0.068 | 0.389 |  |
| Locomotor | <--- | Aesthetics | 0.237 | 0.051 | 0.524 |  | **1.338** | **0.252** | **0.003** |  |
| Locomotor | <--- | Cycling | 0.356 | 0.05 | 0.664 |  | **3.36** | **0.436** | ******* |  |
| Locomotor | <--- | Walking | 0.257 | 0.03 | 0.804 |  | -1.919 | -0.216 | 0.097 |  |
| Locomotor | <--- | Sport Play | 0.196 | 0.041 | 0.682 |  | 0.174 | 0.03 | 0.747 |  |
| Locomotor | <--- | Other Facilities | 0.482 | 0.081 | 0.414 |  | 0.765 | 0.117 | 0.236 |  |
| Locomotor | <--- | Design | 0.063 | 0.007 | 0.935 |  | 0.029 | 0.003 | 0.975 |  |
| Locomotor | <--- | Age | 0.455 | 0.033 | 0.647 |  | **3.809** | **0.241** | ******* |  |
| Locomotor | <--- | Environment | 0.068 | 0.136 | 0.183 |  | 0.092 | 0.161 | 0.078 |  |
| Locomotor | <--- | PL Self Description | 0.035 | 0.077 | 0.469 |  | -0.074 | -0.133 | 0.127 |  |
| Locomotor | <--- | Rank of Literacy | 0.023 | 0.055 | 0.487 |  | -0.02 | -0.035 | 0.617 |  |
| Locomotor | <--- | Logistics | 2.45 | 0.179 | 0.07 |  | 2.554 | 0.152 | 0.104 |  |
| Locomotor | <--- | Modeling | **-2.987** | **-0.272** | **0.003** |  | 0.473 | 0.028 | 0.741 |  |
| Locomotor | <--- | Community use | 0.085 | 0.006 | 0.95 |  | 1.253 | 0.075 | 0.439 |  |
| Locomotor | <--- | Restricting | 1.247 | 0.089 | 0.291 |  | -0.64 | -0.042 | 0.595 |  |

WD=weekday, WE=weekend, PL=Physical literacy; non-stand=none standardized, bold text marks significant effects with p < 0.05 or more; *** p<0.001.

Table 6. Direct effects of movement behavior, social and physical environment on Upper Body Control, N=304.

|  |  |  | Boys | | |  | Girls | | |  |
| --- | --- | --- | --- | --- | --- | --- | --- | --- | --- | --- |
| Parameter |  | Correlates | non-stand estimate | standardized estimate | *p* | R^2^ | non-stand estimate | standardized estimate | *p* | R^2^ |
| Upper Body control | <--- | Sport time | **0.958** | **0.193** | **0.012** | **0.30** | **0.616** | **0.13** | **0.018** | **0.22** |
| Upper Body control | <--- | Playing outdoor WD | 0.579 | 0.101 | 0.201 |  | 0.267 | 0.049 | 0.392 |  |
| Upper Body control | <--- | Playing outdoor WE | 0.123 | 0.021 | 0.807 |  | 0.217 | 0.04 | 0.531 |  |
| Upper Body control | <--- | Aesthetics | 0.589 | 0.123 | 0.097 |  | **0.551** | **0.12** | **0.042** |  |
| Upper Body control | <--- | Cycling | **-2.015** | **-0.276** | **0.01** |  | -1.02 | -0.147 | 0.083 |  |
| Upper Body control | <--- | Walking | 1.851 | 0.206 | 0.061 |  | **1.85** | **0.217** | **0.009** |  |
| Upper Body control | <--- | Sport Play | -0.645 | -0.129 | 0.157 |  | -0.441 | -0.093 | 0.184 |  |
| Upper Body control | <--- | Other Facilities | 0.615 | 0.1 | 0.275 |  | 0.39 | 0.067 | 0.339 |  |
| Upper Body control | <--- | Design | -0.42 | -0.045 | 0.569 |  | -0.528 | -0.059 | 0.454 |  |
| Upper Body control | <--- | Age | 1.166 | 0.081 | 0.219 |  | 1.093 | 0.08 | 0.114 |  |
| Upper Body control | <--- | Environment | 0.078 | 0.151 | 0.108 |  | **0.116** | **0.235** | ******* |  |
| Upper Body control | <--- | PL Self Description | 0.083 | 0.176 | 0.071 |  | 0.008 | 0.017 | 0.811 |  |
| Upper Body control | <--- | Rank of Literacy | **-0.093** | **-0.217** | **0.003** |  | **-0.085** | **-0.207** | ******* |  |
| Upper Body control | <--- | Logistics | **4.314** | **0.305** | ******* |  | **3.43** | **0.255** | ******* |  |
| Upper Body control | <--- | Modeling | -0.896 | -0.079 | 0.352 |  | -0.654 | -0.06 | 0.386 |  |
| Upper Body control | <--- | Community use | -2.049 | -0.142 | 0.109 |  | -1.303 | -0.095 | 0.178 |  |
| Upper Body control | <--- | Restricting | -0.914 | -0.063 | 0.417 |  | -0.87 | -0.063 | 0.263 |  |

WD=weekday, WE=weekend, PL=Physical literacy; non-stand=none standardized, bold text marks significant effects with p < 0.05 or more; *** p<0.001.

Table 7. Direct effects of movement behavior, social and physical environment on Lower Body Control, N=304.

|  |  |  | Boys | | |  | Girls | | |  |
| --- | --- | --- | --- | --- | --- | --- | --- | --- | --- | --- |
| Parameter |  | Correlates | none-stand estimate | standardized estimate | *p* | R^2^ | none-stand estimate | standardized estimate | *p* | R^2^ |
| Lower Body control | <--- | Sport time | 0.612 | 0.152 | 0.062 | **0.2** | 0.103 | 0.028 | 0.75 | **0.22** |
| Lower Body control | <--- | Playing outdoor WD | **1.026** | **0.221** | **0.009** |  | 0.254 | 0.054 | 0.516 |  |
| Lower Body control | <--- | Playing outdoor WE | -0.602 | -0.128 | 0.163 |  | 0.414 | 0.078 | 0.355 |  |
| Lower Body control | <--- | Aesthetics | 0.316 | 0.081 | 0.301 |  | 0.188 | 0.047 | 0.607 |  |
| Lower Body control | <--- | Cycling | -0.909 | -0.153 | 0.177 |  | 0.07 | 0.012 | 0.929 |  |
| Lower Body control | <--- | Walking | 1.643 | 0.225 | 0.053 |  | 1.384 | 0.207 | 0.137 |  |
| Lower Body control | <--- | Sport Play | -0.658 | -0.162 | 0.094 |  | 0.08 | 0.018 | 0.853 |  |
| Lower Body control | <--- | Other Facilities | 0.163 | 0.033 | 0.736 |  | -0.593 | -0.121 | 0.253 |  |
| Lower Body control | <--- | Design | 0.461 | 0.061 | 0.467 |  | 1.114 | 0.142 | 0.136 |  |
| Lower Body control | <--- | Age | 1.533 | 0.132 | 0.061 |  | **-0.27** | **-0.023** | **0.034** |  |
| Lower Body control | <--- | Environment | 0.03 | 0.072 | 0.471 |  | 0.09 | 0.207 | 0.47 |  |
| Lower Body control | <--- | PL Self Description | 0.065 | 0.169 | 0.102 |  | 0.028 | 0.067 | 0.148 |  |
| Lower Body control | <--- | Rank of Literacy | **-0.081** | **-0.233** | **0.003** |  | **-0.047** | **-0.108** | **0.009** |  |
| Lower Body control | <--- | Logistics | 1.981 | 0.172 | 0.075 |  | 3.28 | 0.259 | 0.258 |  |
| Lower Body control | <--- | Modeling | 0.068 | 0.007 | 0.935 |  | -1.299 | -0.102 | 0.658 |  |
| Lower Body control | <--- | Community use | -0.379 | -0.032 | 0.731 |  | -0.577 | -0.046 | 0.92 |  |
| Lower Body control | <--- | Restricting | -1.295 | -0.109 | 0.182 |  | -0.097 | -0.008 | 0.88 |  |

WD=weekday, WE=weekend, PL=Physical literacy; non-stand=none standardized, bold text marks significant effects with p < 0.05 or more.

Table 8. Direct effects of movement behavior, social and physical environment on Balance, N=304.

|  |  |  | Boys |  |  |  | Girls |  |  |  |
| --- | --- | --- | --- | --- | --- | --- | --- | --- | --- | --- |
| Parameter |  | Correlates | none-stand estimate | standardized estimate | *p* | R^2^ | none-stand estimate | standardized estimate | *p* | R^2^ |
| Balance | <--- | Sport time | **0.81** | **0.172** | **0.026** | **0.3** | -0.105 | -0.028 | 0.752 | **0.2** |
| Balance | <--- | Playing outdoor WD | 0.77 | 0.142 | 0.075 |  | 0.167 | 0.035 | 0.678 |  |
| Balance | <--- | Playing outdoor WE | 0.23 | 0.042 | 0.63 |  | 0.435 | 0.081 | 0.342 |  |
| Balance | <--- | Aesthetics | **1.154** | **0.254** | ******* |  | **0.845** | **0.208** | **0.024** |  |
| Balance | <--- | Cycling | **-2.191** | **-0.317** | **0.003** |  | 0.814 | 0.138 | 0.315 |  |
| Balance | <--- | Walking | **2.363** | **0.278** | **0.012** |  | 0.993 | 0.147 | 0.297 |  |
| Balance | <--- | Sport Play | -0.372 | -0.079 | 0.392 |  | **-1.245** | **-0.284** | **0.005** |  |
| Balance | <--- | Other Facilities | **2.014** | **0.347** | ******* |  | **1.597** | **0.32** | **0.003** |  |
| Balance | <--- | Design | 0.048 | 0.005 | 0.946 |  | -0.459 | -0.058 | 0.548 |  |
| Balance | <--- | Age | 0.315 | 0.023 | 0.728 |  | **2.119** | **0.175** | **0.019** |  |
| Balance | <--- | Environment | 0.042 | 0.086 | 0.365 |  | 0.003 | 0.007 | 0.945 |  |
| Balance | <--- | PL Self Description | 0.041 | 0.093 | 0.346 |  | 0.03 | 0.07 | 0.456 |  |
| Balance | <--- | Rank of Literacy | **-0.061** | **-0.149** | **0.042** |  | -0.013 | -0.03 | 0.687 |  |
| Balance | <--- | Logistics | **2.427** | **0.181** | **0.049** |  | 1.56 | 0.122 | 0.226 |  |
| Balance | <--- | Modeling | -0.808 | -0.075 | 0.379 |  | -0.403 | -0.031 | 0.731 |  |
| Balance | <--- | Community use | -1.273 | -0.093 | 0.297 |  | 0.924 | 0.073 | 0.489 |  |
| Balance | <--- | Restricting | -0.11 | -0.008 | 0.918 |  | -1.671 | -0.143 | 0.091 |  |

WD=weekday, WE=weekend, PL=Physical literacy; non-stand=none standardized, bold text marks significant effects with p < 0.05 or more; *** p<0.001.

Table 9. Direct effects of movement behavior, social and physical environment on Running, N=304.

|  |  |  | Boys | | |  | Girls | | |  |
| --- | --- | --- | --- | --- | --- | --- | --- | --- | --- | --- |
| Parameter |  | Correlates | none-stand Estimate | standardized estimate | *p* | R^2^ | none-stand Estimate | standardized estimate | *p* | R^2^ |
| Running | <--- | Sport time | -0.023 | -0.007 | 0.938 | **0.2** | -0.12 | -0.034 | 0.705 | **0.14** |
| Running | <--- | Playing outdoor WD | **0.727** | **0.182** | **0.037** |  | -0.179 | -0.04 | 0.641 |  |
| Running | <--- | Playing outdoor WE | 0.276 | 0.068 | 0.472 |  | 0.531 | 0.106 | 0.226 |  |
| Running | <--- | Aesthetics | -0.002 | -0.001 | 0.993 |  | 0.338 | 0.09 | 0.346 |  |
| Running | <--- | Cycling | -0.943 | -0.185 | 0.116 |  | **1.66** | **0.304** | **0.032** |  |
| Running | <--- | Walking | 1.379 | 0.22 | 0.069 |  | -1.044 | -0.166 | 0.253 |  |
| Running | <--- | Sport Play | -0.118 | -0.034 | 0.737 |  | -0.488 | -0.12 | 0.249 |  |
| Running | <--- | Other Facilities | 0.257 | 0.06 | 0.552 |  | 0.533 | 0.115 | 0.295 |  |
| Running | <--- | Design | 0.666 | 0.102 | 0.239 |  | 0.582 | 0.079 | 0.427 |  |
| Running | <--- | Age | 0.188 | 0.019 | 0.796 |  | 1.343 | 0.12 | 0.121 |  |
| Running | <--- | Environment | **0.075** | **0.207** | **0.044** |  | 0.001 | 0.002 | 0.988 |  |
| Running | <--- | PL Self Description | -0.017 | -0.053 | 0.623 |  | 0.002 | 0.005 | 0.955 |  |
| Running | <--- | Rank of Literacy | -0.002 | -0.007 | 0.93 |  | **-0.065** | **-0.16** | **0.04** |  |
| Running | <--- | Logistics | **2.155** | **0.218** | **0.03** |  | **3.105** | **0.261** | **0.012** |  |
| Running | <--- | Modeling | **-1.574** | **-0.198** | **0.033** |  | -0.524 | -0.044 | 0.642 |  |
| Running | <--- | Community use | 0.831 | 0.082 | 0.397 |  | 0.055 | 0.005 | 0.966 |  |
| Running | <--- | Restricting | -0.719 | -0.071 | 0.405 |  | -0.45 | -0.042 | 0.635 |  |

WD=weekday, WE=weekend, PL=Physical literacy; non-stand=none standardized, bold text marks significant effects with p < 0.05 or more.
